# Impact of blood pressure changes on myocardial work indices in hypertensive patients in a day

**DOI:** 10.1111/jch.14379

**Published:** 2021-12-10

**Authors:** Xinhao Li, Quande Liu, Wuyun Bao, Mengmeng Li, Yu Zhang, Xiaoyu Wan, Mei Zhang

**Affiliations:** ^1^ The Key Laboratory of Cardiovascular Remodeling and Function Research Chinese Ministry of Education Chinese National Health Commission and Chinese Academy of Medical Sciences The State and Shandong Province Joint Key Laboratory of Translational Cardiovascular Medicine Department of Cardiology Qilu Hospital Cheeloo College of Medicine Shandong University Shandong China

**Keywords:** blood pressure, echocardiography, global longitudinal strain, hypertension, myocardial work

## Abstract

Evaluating left ventricular function through instantaneous left ventricular deformation parameters might not always be accurate for patients with high fluctuations in blood pressure value due to afterload dependence. Myocardial work (MW) is a more advanced tool that combines global myocardial longitudinal strain (GLS) with LV (left ventricular) systolic pressure. The purpose of this study was to investigate the effect of blood pressure changes on MW indices in the population with normal blood pressure and hypertension in a day. A total of 117 participants (34 control subjects and 83 hypertensive patients) underwent echocardiographic measurements at rest, twice a day. Simultaneously, the brachial blood pressure was also measured. LV pressure‐strain loop (PSL) was used to calculate global work index (GWI), global constructive work (GCW), global wasted work (GWW), and global work efficiency (GWE). The differences in the GLS and MW indices between the groups were compared, and the correlation of blood pressure changes with the changes in GLS and MW indices were evaluated. Compared to the control group, the hypertensive group showed higher GWI, GCW, and GWW but lower GLS and GWE. Absolute changes in blood pressure, GLS, and MW indices in hypertensive patients were significantly higher than that of the control subjects. Blood pressure changes had significant univariate correlation with changes in GLS and MW indices. In conclusion, significant fluctuations in blood pressure could induce changes in MW indices to preserve left ventricular systolic function. Repeated assessment of MW indices is necessary for hypertensive patients with large blood pressure fluctuations.

## 1 INTRODUCTION

Echocardiography is undoubtedly a valuable method in evaluating the left ventricular systolic function in patients with hypertension.^1^ A two‐dimensional (2D) speckle‐tracking echocardiography (STE) is one of the most outstanding innovations in the field of echocardiography in recent decades. Compared to the traditional echocardiographic parameters, using 2D‐STE for myocardial deformation imaging could provide more information about the myocardial function. The Global myocardial longitudinal strain (GLS) derived from 2D‐STE is considered superior to the left ventricular ejection fraction (LVEF) in predicting cardiovascular mortality.^2^, ^3^, ^4^ Furthermore, GLS is associated with left ventricular structural remodeling in patients with hypertension.^5^ Hence, GLS has been regarded as a reliable, sensitive, and reproducible tool for evaluating the left ventricular systolic function.^6^

It is well known that due to the interaction of different mechanisms in response to external and internal stimuli, blood pressure shows spontaneous fluctuation within 24 hours.^7^ These oscillations are documented as a physiological phenomenon, especially in patients with hypertension.^8^, ^9^ However, one of the main limitations of GLS is load dependence, which may affect the accuracy of diagnosis.^10^ It is challenging to distinguish the actual myocardial dysfunction from cardiac functional changes associated with altered load states in hypertensive patients with high blood pressure variability (BPV).

The concept of “Myocardial work” was once put forward by Suga in 1979.^11^ However, due to its invasiveness and complexity, it was not applied widely in clinical practice. Yet, recent studies have confirmed a good correlation and consistency between the invasive and noninvasive left ventricular pressure‐strain loop (LV‐PSL).^12^, ^13^ With the replacement of noninvasive LV‐PSL, applying myocardial work (MW) in daily practice has become more feasible. MW is considered as an advancement of GLS, which combines deformation as well as afterload. MW measures the amount of work performed starting from the closure of the mitral valve till its opening, which is an indirect measurement of myocardial metabolism, systolic stroke work, and oxygen consumption.^12^ The Normal Reference Ranges for Echocardiography (NORRE) study by Manganaro and associates provides normal reference limits for MW indices.^14^ As a part of their study, Manganaro and associates also found that MW indices showed no strong correlation with age and sex.^14^ Currently, MW has been used to evaluate left ventricular systolic function under different clinical conditions. In a series of studies, Galli and associates demonstrated that MW may be used as a reliable predictor of response to cardiac resynchronization therapy.^15^, ^16^, ^17^ Also, few other studies have shown that MW diagnoses not only hemodynamically significant coronary stenosis in stable coronary arteries but also identifies acute coronary occlusion in patients with non‐ST‐segment elevation acute coronary syndrome (NSTE‐ACS).^18^, ^19^ Moreover, the evaluation of MW for other cardiovascular diseases is also going on in full swing. However, to our knowledge, no research has explored the differences in MW indices under different blood pressure conditions in resting state. Hence, the purpose of this article was to investigate the effect of blood pressure changes on MW indices in participants with normal blood pressure and hypertension in a day.

## 2 METHODS

### 2.1 Study cohort

This was a single‐center, prospective study that recruited hypertensive patients referred for echocardiography. The exclusion criteria included: (a) suboptimal image quality; (b) left ventricular ejection fraction (LVEF) < 50%; (c) atrial fibrillation or other severe arrhythmias; (d) moderate or severe valvular stenosis; (e) intracardiac shunt. Four patients having systolic blood pressure ≥ 180 mmHg or diastolic blood pressure ≥ 110 mmHg with no associated symptoms were treated immediately with nifedipine, while the others did not take any drugs before and during the test. After the test, patients were treated with antihypertensive drugs according to their blood pressure values. Patients with systolic blood pressure ≥ 180 mmHg or diastolic blood pressure ≥ 110 mmHg displaying associated symptoms were not included in the study. Based on the selection criteria, we enrolled healthy controls without structural heart disease or cardiovascular risk factors and hypertensive patients. The study was approved by the Ethics Committee of Scientific Research of Shandong University Qilu Hospital (KYLL‐202011–100) and conducted as per the Declaration of Helsinki. All patients and control subjects were informed about the study. According to the 2020 International Society of Hypertension (ISH) and Global Hypertension Practice Guidelines, the hypertensive patients were subdivided into Grade 1 hypertension (140–159/90–99 mmHg) and Grade 2 hypertension (≥160/100 mmHg).^20^

### 2.2 Acquisition of echocardiographic and blood pressure data

Transthoracic echocardiographic imaging was obtained using a Vivid E95 machine equipped with an M5S 3.5 MHz transducer (GE, Vingmed Ultrasound, Horten, Norway). Echocardiography of patients was performed by the same experienced sonographers twice a day between 9 am and 5 pm with an interval of over 4 hours. The patients were at rest with left side decubitus. The standard imaging windows and measurements were obtained according to the current guidelines of the American Society of Echocardiography and the European Association of Cardiovascular Imaging.^6^, ^21^ Images were stored for at least three cardiac cycles.

The cardiac structural and functional measures included left atrium longitudinal dimension (LA‐l), right atrium longitudinal dimension (RA‐l), interventricular septal end‐diastolic dimension (IVSd), right ventricular end‐diastolic dimension (RVDd), left ventricular diameter diastole (LVDd), left ventricular end‐diastolic posterior wall thickness (PWTd), relative wall thickness (RWT), left atrium volume index (LAVI), left ventricular mass index (LVMI), late mitral inflow velocity (A), early mitral inflow velocity (E), peak early diastolic mitral annular velocity (e’), and left ventricular ejection fraction (LVEF). The LVEF was calculated using the biplane Simpson's method while the Left ventricular mass (LVM) was derived from the Devereux formula, and the left atrial volume (LAV) was measured at the LV end‐systole. LVMI and LAVI were obtained by correcting the LVM and LAV through body surface area (BSA). According to the 2018 ESC/ESH Guidelines for the management of arterial hypertension, LV hypertrophy (LVH) was defined as LVMI > 115 g/m^2^ in men and LVMI > 95 g/m^2^ in women. LV geometry was categorized as follows: (a) Normal geometry: no LVH and RWT < 0.43; (b) Concentric remodeling: no LVH and RWT ≥0.43; (c) Eccentric hypertrophy: LVH and RWT < 0.43; (d) Concentric hypertrophy: LVH and RWT ≥0.43.^8^

Simultaneous with echocardiography, blood pressure values were measured on the right brachial artery using an automated blood pressure monitor (Omron 7200, Omron Healthcare). Smoking and drinking coffee, tea, or alcohol and exercise were prohibited for 30 minutes before measuring BP. Patients did not fast for lunch but were asked to eat a light and not a full meal. Before the procedure, patients were asked to empty their bladder and relax for 3–5 minutes. During the BP measurements, the patients remained quiet and were in the supine position. The BP measurements were taken thrice with 1–2 minute intervals, and the average value was used for the analyses.^20^ Mean arterial blood pressure (MBP) was calculated as one‐third of the systolic blood pressure (SBP) plus two‐third of the diastolic blood pressure (DBP). Further, SBP was compared in the morning and afternoon and the smaller value was selected as the baseline.

### 2.3 Two‐dimensional STE

To evaluate the GLS using STE, the standard imaging windows were acquired from the three apical views (the apical four‐chamber, two‐chamber, and long axis) at frame rates between 50 and 80 frames/s. The myocardial motion in the region of interest was automatically tracked using the Automated Function Imaging software (EchoPAC Version 203). If necessary, the region of interest was adjusted by correcting the edge or width of the endocardium. According to the standardized 17‐segment heart model,^22^ GLS was calculated from the mean of the longitudinal peak systolic strain of all the LV segments. Also, all GLS values were reported using absolute values.

### 2.4 Quantification and analysis of myocardial work

MW was quantified by the noninvasive PSL, which integrated the brachial blood pressure into the LV strain parameters using the EchoPAC software. The area inside the PSL served as an indicator of the myocardial work index. In the process of LV ejection, the constructive and waste work of the myocardial segments were analyzed, and the global values were calculated as an average of all segment values.

The closing and opening times of the aortic and mitral valves were measured through the apical long‐axis view using 2D‐echocardiography. Based on the LV strain, brachial blood pressure, and the valvular event times, the following LV myocardial work parameters were calculated by the software:

Global myocardial work index (GWI, mmHg%): the myocardial work, expressed as the area of LV‐PSL, was calculated starting from the closure of the mitral valve till the opening of the mitral valve.

Global constructive myocardial work (GCW, mmHg%): the “positive” work performed by the LV segments contributing to the LV ejection (shortening during systole and lengthening during isovolumic relaxation).

Global wasted myocardial work (GWW, mmHg%): the “negative” work performed by the LV segments not contributing to the LV ejection (lengthening during systole and shortening during isovolumic relaxation).

Global myocardial work efficiency (GWE, %): the percentage of myocardial work, calculated as the ratio of GCW to the sum of GCW and GWW.

### 2.5 Intra‐ and inter‐observer variability

Two experienced sonographers re‐measured 30 randomly selected participants to assess the repeatability. In the process, sonographers were blinded to the clinical data as well as to each other's results. A month later, the images were analyzed again by the same sonographer to assess the intra‐observer variability. The same images were also analyzed by both sonographers to assess the inter‐observer variability.

### 2.6 Statistical analysis

All data were collected, statistically analyzed, and tabulated using the SPSS 25 software (SPSS Inc., Chicago, IL, USA) and Med Calc 19.04 (Med Calc Software BVBA, Ostend, Belgium). Visual checks and the Shapiro‐Wilk test were applied to test normality. Continuous variables were presented as mean ± SD or mean± SE. Categorical data variables were shown as number (n) and percentage (%). The Chi‐square test was used to compare the categorical data. For continuous data, the Student's *t*‐test was used to compare the data between two groups, while one‐way analysis of variance (ANOVA) with Tukey's honestly significant difference (HSD) post‐hoc tests (for equal variances) or Games‐Howell HSD post‐hoc tests (for unequal variances) were performed to compare multigroup data. Analysis of covariance and multivariable linear regression was used to adjust the influence of confounding variables. Correlations between the parameters were assessed by Pearson's correlation test. All tests were two‐tailed with 95% confidence intervals (CI), and *P‐*values < .05 were considered statistically significant. Intra‐class correlation coefficients (ICC) and Bland‐Altman plots were used to assess the inter‐and intra‐observer variability.

## 3 RESULTS

### 3.1 Demographic data

The study cohort included 34 control subjects with no history of hypertension and 83 hypertensive patients, of whom 45 (54.22%) had Grade 1 hypertension and 38 (45.78%) had Grade 2 hypertension. Patients with hypertension tended to have higher age, body mass index, and body surface area (all *P* < .05). No significant inter‐group differences were observed in the heart rate (HR), sex, and history of diabetes. In terms of cardiac structure, hypertensive patients were more likely to have larger dimensions of the atrium, ventricle, and interventricular septal along with higher LAVI and bigger LVMI (all *P* < .05). Regarding the left ventricular function, hypertensive groups displayed significantly higher E/e’ but showed lower E/A and LVEF (all *P* < .05). Compared to the controls, hypertensive patients showed a higher percentage of LVH, eccentric hypertrophy, and concentric hypertrophy. The clinical characteristics of the study participants are shown in Table 1.

**TABLE 1:** Characteristics of the healthy control subjects and patients with hypertension

		HTN		
	Controls (no. = 34)	Grade 1 (no. = 45)	Grade 2 (no. = 38)	*P* (overall)
Age (years)	44.76±12.77	55.58±10.57*	58.50±9.70*	<.001
Men, no. (%)	19(55.88%)	25(55.56%)	20(52.63%)	.098
HR (b.p.m.)	72.62±7.63	67.24±9.86	70.45±11.90	.060
BSA(m^2^)	1.71±0.15	1.79±0.17	1.82±0.17*	.010
BMI (kg/m^2^)	21.29±2.48	26.06±3.10*	26.88±3.20*	<.001
Diabetes	0 (0.00%)	4(8.89%)	5(13.16%)	.094
LA‐l (mm)	41.68±6.37	48.96±6.95*	50.05±5.28*	<.001
RA‐l (mm)	38.09±5.46	42.13±6.16*	41.68±5.00*	.040
IVSd (mm)	9.03±1.38	11.67±1.64*	12.03±1.75*	<.001
LVDd (mm)	43.09±3.03	45.96±5.15*	47.18±4.84*	.001
PWTd (mm)	9.15±1.16	9.47±1. 27	10.18±1.43* ^†^	.003
RVDd (mm)	25.32±4.03	29.20±3.46*	29.16±4.16*	<.001
RWT	0.43±0.05	0.42±0.08	0.44±0.07	.475
LAVI (ml/m^2^)	20.88±4.65	28.57±9.14*	31.95±9.83*	<.001
LV mass index (g/m^2^)	73.66±8.90	96.52±18.71*	104.19±17.07*	<.001
LV hypertrophy	0 (0.00%)	15(33.33%)	18(47.27%)	<.001
LV geometry				
Normal geometry	28(82.35%)	18(40.00%)	7(18.42%)	<.001
Concentric remodeling	6(17.65%)	12(26.67%)	13(34.21%)	.280
Eccentric hypertrophy	0 (0.00%)	8(17.78%)	7(18.42%)	.013
Concentric hypertrophy	0 (0.00%)	7(15.55%)	11(28.94%)	.003
E/A	1.64(1.40‐1.96)	0.82(0.70‐0.96) *	0.73(0.52‐0.96) *	<.001
E/e’ (cm/s)	6.08(5.40‐7.54)	8.00(6.54‐9.33) *	9.20(7.14‐12.51) *	<.001
LV ejection fraction (%)	68.91±3.78	66.02±6.30*	64.61±6.07*	.005

Abbrevaitions: HTN, hypertension; HR, heart rate; BSA, body surface area; BMI, body mass index; LA‐l, left atrium longitudinal dimension; RA‐l, right atrium longitudinal dimension; IVSd, interventricular septal end‐diastolic dimension; LVDd, left ventricular diameter diastole; PWTd, left ventricular end‐diastolic posterior wall thickness; RVDd, right ventricular end‐diastolic dimension; RWT, relative wall thickness; LAVI, left atrium volume index; LV, left ventricular; E, peak early diastolic mitral flow velocity; A, late diastolic mitral flow velocity; e′, peak early diastolic mitral annular velocity; LVEF, LV ejection fraction.

* *P* < .05 vs control group by post‐hoc tests. ^†^ *P* < .05 vs grade 1 group by post‐hoc tests.

### 3.2 GLS and MW analysis

The GWI, GCW, and GWW were found to be higher in the hypertensive groups, while GLS and GWE were found to be lower compared to the control group (all *P* < .05). Among hypertensive patients, GWI, GCW, and GWW were higher in Grade 2 group than that in the Grade 1 group. However, GLS and GWE did not reach significant differences within the hypertensive groups. With the adjustments applied to age, BSA, and BMI at the baseline, no obvious difference was observed in GLS between hypertensive patients and the controls. All information is presented in Tables 2 and 3.

**TABLE 2:** Comparison of GLS and MW indices between the participants with higher SBP state and baseline SBP state

	Groups	Controls(no. = 34)	*P*	HTN Grade 1 (no. = 45)	*P*	HTN Grade 2 (no. = 38)	*P*	*P* (overall)
SBP	higher SBP	119.53±10.99	<.001	148.78±4.90*	<.001	169.13±8.21* ^†^	<.001	<.001
	baseline SBP	114.00±9.26		131.69±8.75*		145.42±14.06* ^†^		<.001
ΔSBP		5.00(3.00‐8.00)		16.00(9.50‐22.50) *		23.00(14.50‐32.50) *		<.001
DBP	higher SBP	71.88±7.16	.011	83.29±8.45*	<.001	97.21±10.38* ^†^	<.001	<.001
	baseline SBP	69.59±7.64		75.84±7.28*		87.16±11.50* ^†^		<.001
ΔDBP		2.00(‐1.00‐5.00)		7.00(3.00‐11.50) *		10.00(3.75‐14.25) *		<.001
MBP	higher SBP	87.76±7.67	<.001	105.12±5.85*	<.001	121.18±8.23* ^†^	<.001	<.001
	baseline SBP	84.47±7.34		94.46±6.12*		106.58±11.32* ^†^		<.001
ΔMBP		2.33(1.08‐5.75)		10.67(5.17‐14.67) *		12.50(7.67‐20.33) *		<.001
GLS	higher SBP	19.04±1.81	.076	17.19±2.05*	<.001	16.90±1.49*	<.001	<.001
	baseline SBP	19.34±1.83		18.03±1.87*		17.91±1.66*		.001
ΔGLS		‐0.30 ±0.95		‐0.84 ±1.17*		‐1.01±0.77*		.008
GWI	higher SBP	1722.18 ±210.69	.197	2010.33±299.65*	<.001	2228.00±301.12* ^†^	<.001	<.001
	baseline SBP	1687.88±206.46		1856.44±224.14*		1981.55±293.47*		<.001
ΔGWI		34.29 ±151.96		153.89 ±247.47*		246.45 ±167.94*		<.001
GCW	higher SBP	1988.12±217.88	.007	2295.71±289.02*	<.001	2623.13±308.18* ^†^	<.001	<.001
	baseline SBP	1921.76±215.47		2093.56±220.24*		2311.11±303.26* ^†^		<.001
ΔGCW		61.50(‐7.00‐140.00)		184.00(35.50 ‐347.00) *		278.50(162.75‐450.50) *		<.001
GWW	higher SBP	78.50(49.75‐99.00)	.300	144.00(104.00‐210.00) *	<.001	199.50(153.50‐253.00) * ^†^	<.001	<.001
	baseline SBP	65.00(52.25‐84.00)		105.00(77.00‐145.50) *		132.50(97.50‐181.00) *		<.001
ΔGWW		3.50(‐11.25‐15.50)		44.00(23.00‐72.50) *		59.00(34.75‐81.75) *		<.001
GWE	higher SBP	96.30(95.34‐97.63)	.739	94.21(91.32‐95.72) *	<.001	93.28(91.19‐94.24) *	<.001	<.001
	baseline SBP	96.30(95.62‐97.36)		95.14(93.76‐96.35) *		94.29(91.83‐95.68) *		<.001
ΔGWE		0.03(‐0.81‐0.55)		‐1.16 (‐2.43‐ ‐0.72) *		‐1.38(‐2.19‐ ‐0.78) *		<.001

Abbreviations: HTN, hypertension; SBP, systolic blood pressure; DBP, diastolic blood pressure; MBP, mean arterial blood pressure; GLS, global longitudinal strain; GWI, global myocardial work index; GCW, global constructive myocardial work; GWW, global wasted myocardial work; GWE, global myocardial work efficiency.

* *P* < .05 vs control group by post‐hoc tests. ^†^ *P* < .05 vs grade 1 group by post‐hoc tests. Δ, parameters value of higher SBP state minus that of baseline SBP state.

**TABLE 3:** Comparison of GLS and MW indices between participants with higher SBP state and baseline SBP state with an adjustment applied for age, BMI, and BSA

	Groups	Controls(no. = 34) adjust mean ± SE	HTN Grade 1 (no. = 45) adjust mean ± SE	HTN Grade 2 (no. = 38) adjust mean ± SE	*P* (overall)
GLS	higher SBP	18.96±0.41	17.21±0.29*	16.94±0.33*	.002
	baseline SBP	19.17±0.41	18.07±0.28	18.01±0.33	.098
ΔGLS		‐0.21 ±0.22	‐0.85 ±0.15	‐1.07±0.18*	.028
GWI	higher SBP	1748.74 ±62.57	2004.01±43.40*	2211.72±50.45* ^†^	<.001
	baseline SBP	1712.18±55.05	1848.73±38.19	1968.95±44.39*	.005
ΔGWI		36.56 ±44.54	155.28±30.89	242.77 ±35.91*	.007
GCW	higher SBP	1970.36±62.62	2301.56±43.43*	2632.09±50.49* ^†^	<.001
	baseline SBP	1901.12±56.17	2098.59±38.96*	2323.61±45.29* ^†^	<.001
ΔGCW		69.23±45.73	202.97±31.72	308.48±36.87*	.002
GWW	higher SBP	83.85±14.56	158.30±10.10*	211.20±11.74* ^†^	<.001
	baseline SBP	82.73±12.13	110.64±8.41	149.41±9.78*	<.001
ΔGWW		1.12±8.34	47.66±5.78*	61.80±6.72 *	<.001
GWE	higher SBP	95.95±0.54	93.50±0.37*	92.64±0.43*	<.001
	baseline SBP	95.87±0.45	95.00±0.31	94.12±0.36*	.021
ΔGWE		‐0.12±0.34	‐1.30±0.23 *	‐1.46±0.27*	<.001

Values were adjusted for age, BMI, and BSA.

Abbreviations: HTN, hypertension; SBP, systolic blood pressure; DBP, diastolic blood pressure; MBP, mean arterial blood pressure; MW, myocardial work; GLS, global longitudinal strain; GWI, global myocardial work index; GCW, global constructive myocardial work; GWW, global wasted myocardial work; GWE, global myocardial work efficiency.

* *P* < .05 vs control group by post‐hoc tests. ^†^ *P* < .05 vs grade 1 group by post‐hoc tests. Δ, parameters value of higher SBP state minus that of baseline SBP state.

Among hypertensive groups, the values of GWI, GCW, and GWW were higher in the patients with higher SBP state than those in the baseline SBP, while the values of GLS and GWE were lower than those of the baseline SBP (all *P* < .05). Among controls, only GCW was greater in a higher SBP state compared to the baseline SBP (Table 2).

The absolute changes in the LV afterload‐associated variables (SBP, DBP, MBP), GLS, and MW indices in hypertensive groups were significantly higher than those of the control group. However, no significant differences were observed in the changes of GLS and MW indices within the hypertensive groups (Table 2). When adjusted for age, BSA, and BMI, the absolute changes in the GLS and MW indices were significantly higher in the Grade 2 group than those found in the control group. However, in the Grade 1 group, only the changes in GWW and GWE were greater compared to the control group. (Table 3).

### 3.3 Noninvasive LV‐PSL

**FIGURE 1:**
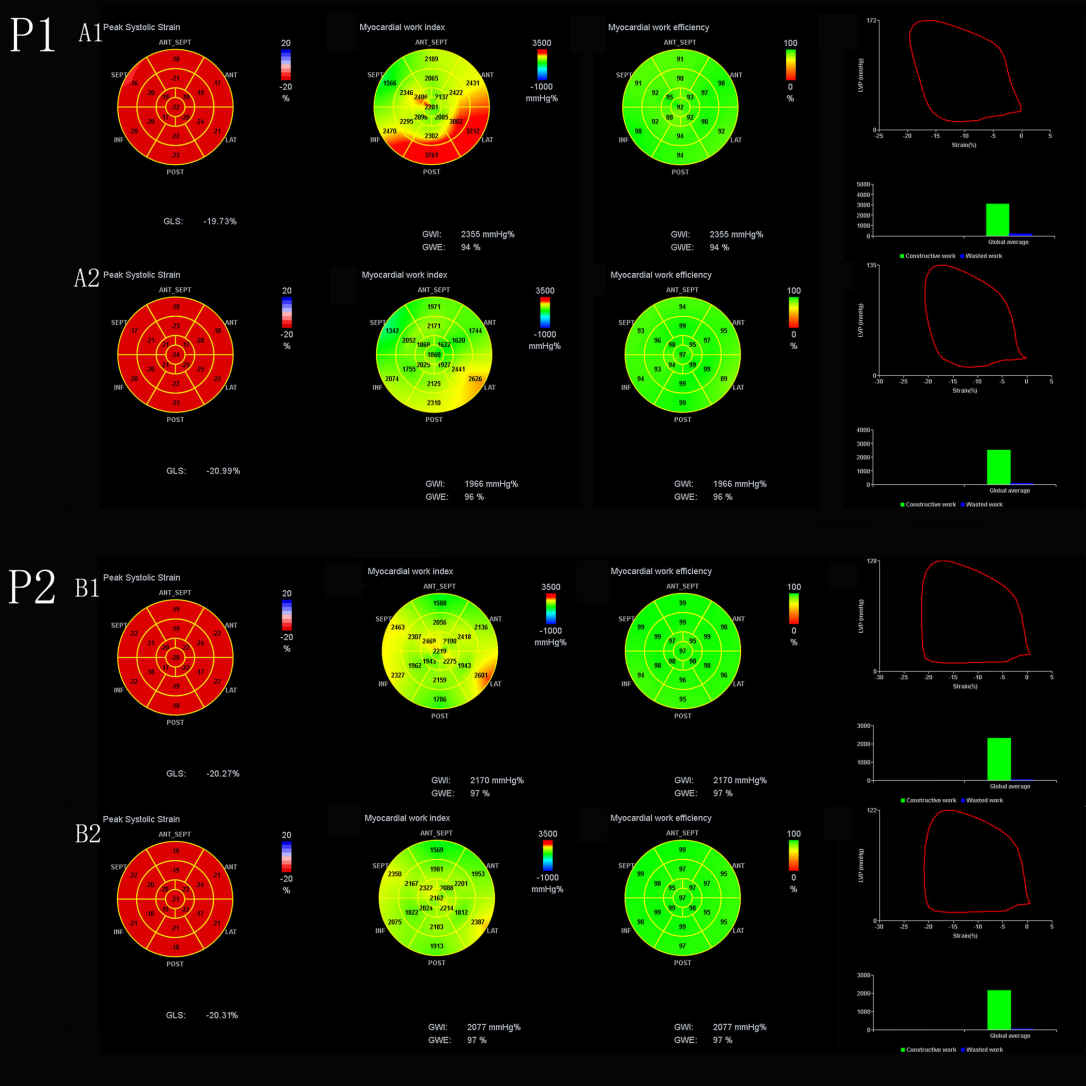
Variation of myocardial work indices and GLS in two participants with two divergent LV afterload at rest. P1, a subject from the control group; P2, a subject from the hypertensive group. P1: 17‐segment bull's‐eye illustrated homogenous myocardial work indices and GLS across all segments at two divergent LV afterloads (A1, BP 128/69 mmHg; A2, BP 122/65 mmHg). P2: Compared to B1, the area of the red segment of bull's‐eye was found to be reduced while the corresponding GLS and GWE were shown to be improved in the B2 (B1, BP 172/92 mmHg; B2, BP 135/80 mmHg)

Figure 1 shows the difference of PSL in the same subject at different afterloads. In participant 2, a decrease in systolic blood pressure shortened the PSL. Furthermore, as the area bound by the PSL decreased, the area of red segments also reduced. In contrast to the reduced GWI and GCW, GLS and GWE values were shown to be elevated (19.73 vs 20.99 for GLS, 94% vs 96% for GWE).

### 3.4 Relationship of LV afterload with GLS and MW indices

**FIGURE 2:**
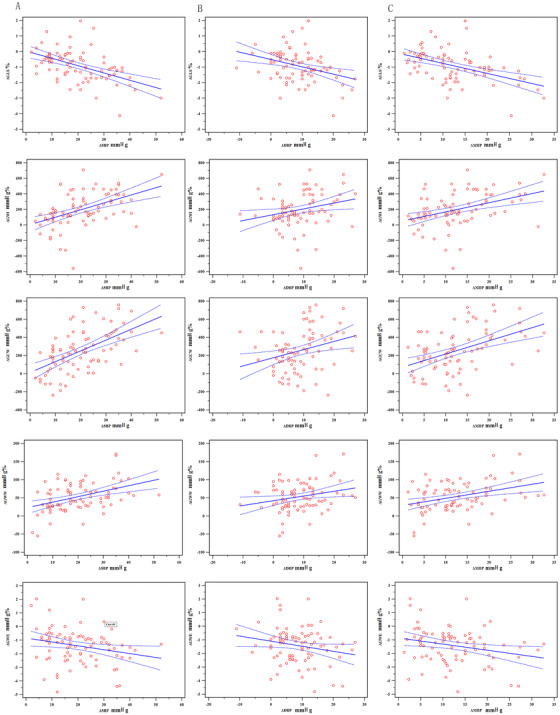
Scatterplots and multivariable linear regression results demonstrating the relationship between blood pressure changes and changes in GLS and MW indices in hypertensive patients. (A), for SBP; (B), for DBP; (C), for MBP

Figure 2 presents the scatterplots and multivariable linear regression results showing the correlation of changes in LV afterload‐associated variables with changes in MW indices and GLS in hypertensive patients. The changes in SBP showed the strongest correlations with changes in GLS, GWI, GCW, and GWW compared to DBP and MBP (r  = ‐0.502 for GLS, r  =  0.480 for GWI, r  =  0.562 for GCW, r  =  0.421 for GWW, r  = ‐0.249 for GWE; all *P *< .05) (Table 4, Figure 2). In the control group, the changes in LV afterload‐associated variables did not show a significant relationship with changes in MW indices and GLS.

**TABLE 4:** Correlation between blood pressure changes and changes in GLS and MW indices

	Δ SBP				Δ DBP				Δ MBP			
	Unadjusted		Adjusted		Unadjusted		Adjusted		Unadjusted		Adjusted	
	r	*P*	*r*	*P*	*r*	*P*	*r*	*P*	*r*	*P*	*r*	*P*
Controls (no. = 34)												
Δ SBP					0.343	.047	0.356	.053	0.586	<.001	0.579	.001
Δ DBP	0.343	.047	0.356	.053					0.962	<.001	0.964	<.001
Δ MBP	0.586	<.001	0.597	.001	0.962	<.001	0.963	<.001				
ΔGLS (%)	‐0.159	.369	‐0.125	.453	‐0.090	.611	‐0.094	.566	‐0.124	.484	‐0.117	.478
ΔGWI (mmHg%)	0.279	.110	0.296	.096	0.207	.240	0.207	.242	0.260	.138	0.262	.136
ΔGCW (mmHg%)	0.218	.216	0.204	.259	0.160	.365	0.159	.372	0.201	.253	0.195	.273
ΔGWW (mmHg%)	‐0.068	.704	‐0.103	.574	0.148	.403	0.153	.397	0.108	.542	0.103	.570
ΔGWE (%)	0.126	.476	0.154	.398	‐0.204	.248	‐0.208	.243	‐0.139	.433	‐0.137	.446
HTN groups (no. = 83)												
Δ SBP					0.487	<.001	0.495	<.001	0.814	<.001	0.819	<.001
Δ DBP	0.487	<.001	0.495	<.001					0.904	<.001	0.909	<.001
Δ MBP	0.814	<.001	0.819	<.001	0.904	<.001	0.909	<.001				
ΔGLS (%)	‐0.500	<.001	‐0.502	<.001	‐0.341	.002	‐0.340	.002	‐0.472	<.001	‐0.469	<.001
ΔGWI (mmHg%)	0.469	<.001	0.480	<.001	0.249	.023	0.246	.026	0.396	<.001	0.397	<.001
ΔGCW (mmHg%)	0.558	<.001	0.562	<.001	0.285	.009	0.294	.008	0.463	<.001	0.468	<.001
ΔGWW (mmHg%)	0.410	<.001	0.421	<.001	0.242	.028	0.231	.039	0.362	.001	0.358	<.001
ΔGWE (%)	‐0.235	.032	‐0.249	.021	‐0.208	.059	‐0.188	.086	‐0.254	.021	‐0.245	.023

Values were adjusted for age, BMI, and BSA.

Δ, parameters value of higher SBP state minus that of baseline SBP state.

Abbreviations: GLS, global longitudinal strain; MW, myocardial work; HTN, hypertension; SBP, systolic blood pressure; DBP, diastolic blood pressure; MBP, mean arterial blood pressure; GWI, global myocardial work index; GCW, global constructive myocardial work; GWW, global wasted myocardial work; GWE, global myocardial work efficiency.

### 3.5 Evaluation of intra‐ and inter‐observer variabilities

**FIGURE 3:**
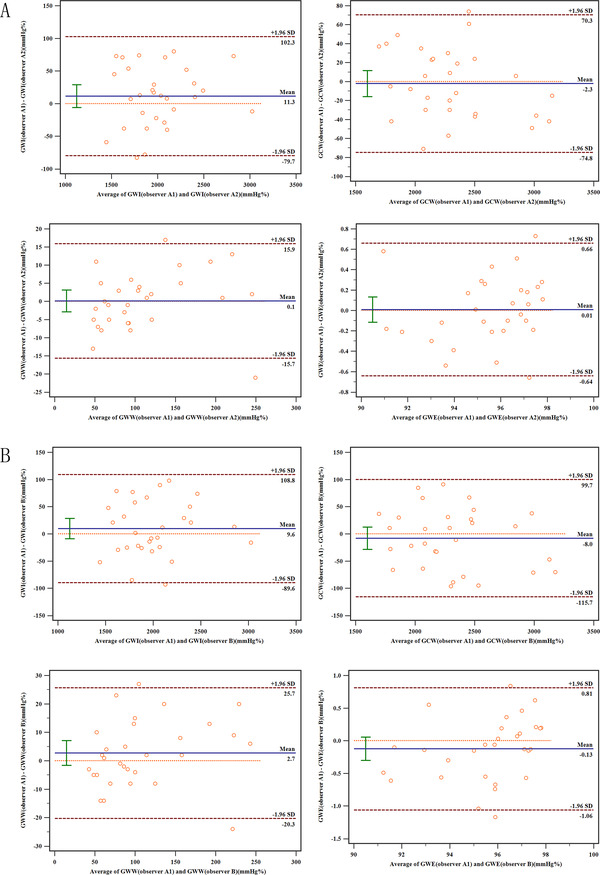
Bland–Altman plots indicating intra‐observer and inter‐observer variabilities in MW indices. The upper and lower dotted lines indicate 95% limits of agreement, while the middle dotted line indicates the zero line. The solid line represents the mean difference between the two measurements. (A), intraobserver variability; (B), interobserver variability

Excellent intra‐observer and inter‐observer variabilities were observed while measuring the MW parameters (Table 5, Figure 3). For the intra‐observer variability, the interclass correlations coefficients (ICC) of GWI, GCW, GWW, and GWE were found to be 0.992, 0.996, 0.991, and 0.986, respectively. For the inter‐observer variability, the ICC of GWI, GCW, GWW, and GWE were found to be 0.990, 0.991, 0.980, and 0.968, respectively.

**TABLE 5:** The intra‐and inter‐observer variabilities of MW indices determined by the interclass correlation coefficients

	Intra‐observer variability			Inter‐observer variability		
	ICC	95%CI	SEM	ICC	95%CI	SEM
GWI (mmHg%)	0.992	0.983‐0.996	247.267	0.990	0.980‐0.995	209.763
GCW (mmHg%)	0.996	0.991‐0.998	465.483	0.991	0.981‐0.996	210.963
GWW (mmHg%)	0.991	0.982‐0.996	221.582	0.980	0.959‐0.991	102.305
GWE (%)	0.986	0.972‐0.993	140.997	0.968	0.935‐0.985	64.264

Abbreviations: CI, confidence interval; ICC, interclass correlation coefficient; SEM, standard error of measurement; MW, myocardial work; GWI, global myocardial work index; GCW, global constructive myocardial work; GWW, global wasted myocardial work; GWE, global myocardial work efficiency.

## 4 DISCUSSION

Our study described the response of MW indices and GLS to the altered blood pressure in hypertensive patients and healthy control subjects. The main findings of this study were as follows: Firstly, in hypertensive patients, a decrease was observed in the GLS while an increase was seen in the GWI with rising blood pressure with no significant changes observed in the controls. Secondly, compared to the controls with smaller LV afterload changes, the absolute changes in GLS and MW indices in hypertensive patients with higher LV afterload changes were higher. Thirdly, a significant correlation was found between the changes in the LV afterload associated variables (SBP, DBP, MBP) and changes in the GLS and MW indices.

Evaluating cardiac systolic function has always been a crucial task in the clinical practice of cardiology. GLS reflects the subendocardial function, which is susceptible to wall stress, ischemia, and fibrosis, through the speckle‐tracking algorithm.^23^ However, numerous studies have confirmed that the increase in afterload was related to the decrease in GLS.^19^, ^24^, ^25^ MW is considered as an advancement of GLS by combining deformation as well as the afterload. Compared to GLS and LVEF, MW can reflect additional cardiac performances in the early stages of the disease. Many studies have confirmed excellent intra‐observer and inter‐observer repeatability of MW indices and results from these studies are also similar to our study.^13^, ^14^, ^24^ The GWI, GCW, GWW, and GWE values were obtained based on the MW analysis. The GWI was referred to the total LV work within the area of the PSL, which was found to have a strong correlation with myocardial glucose metabolism.^12^ The GCW was referred to as the work that contributed to the LV ejection during systole, whereas GWW focused on quantifying lost energy due to the uncoordinated left ventricular contractions. In the recent studies by Galli and associates, GWW and GCW were shown to predict the response of cardiac asynchrony patients to cardiac resynchronization therapy.^15^, ^16^, ^17^ The GWE reflects the efficiency of the mechanical energy consumption during the cardiac cycle. El Mahdiui and associates demonstrated that GWE was found to be similar in normal individuals and also in the ones with the CV risk factors, but in the post‐infarct patients without heart failure and heart failure patients with reduced ejection fraction (HFrEF), it was found to be decreased.^26^ Generally, MW provided more information for a better understanding of the relationship between left ventricular deformation and afterload conditions, which could help us to distinguish the myocardial dysfunction happening from the changes associated with altered afterload.

The left ventricular MW indices have been used for the assessment of patients with hypertension. Chan and associates found that the patients with Grade 2 hypertension showed significantly higher GWI and GCW, while in patients with Grade 1 hypertension, these parameters only tended to increase compared to the controls.^24^ In another study by Jaglan and associates, which used the 2017 American College of Cardiology guidelines, it was proved that GWI was significantly elevated in both Stage 1 and Stage 2 hypertension.^27^ Furthermore, Lembo and associates demonstrated that elevated DBP could not only cause an increase in GWW but also could induce a decrease in GWE.^28^ The results of these studies were consistent with our findings. In our study, we found that compared to the control group, the hypertensive group had higher GWI, GCW, and GWW but lower GLS and GWE. We further examined the difference by adjusting the age, BMI, and BSA. At the baseline state, no significant difference was found in the GLS between hypertensive patients and the control participants, which proved that the GLS might not always be accurate in assessing the left ventricular function, especially in individuals with greater fluctuations of afterload.

Despite MW having great potential for clinical applications, only a few studies have explored the effect of blood pressure fluctuations on myocardial work within one day. We observed an interesting phenomenon in the study, wherein for hypertensive patients, a rise in blood pressure led to a decrease in the GLS and an increase in GWI within one day. A possible explanation for this contradictory phenomenon might be a compensatory mechanism. Firstly, a significant increase in LV afterload might have led to increased wall stress and reduced deformation. Low curvature and high fiber stress make the longitudinal deformation very sensitive to the changes in blood pressure.^29^ Secondly, due to the increased afterload, a short‐term decline in the LV stroke volume may be observed, which could be compensated by increasing GWI while transferring the LV pumps to a higher energy level.^24^, ^29^ However, in controls, no significant changes were observed in the GLS and GWI with elevated afterload. In other words, in the healthy population, GLS and GWI were relatively insusceptible to the changes in the LV afterload within a stable physiological range. This phenomenon provides us with new insights for better distinguishing the actual myocardial dysfunction occurring from changes associated with altered afterload.

Additionally, we found that GCW became significantly greater with an increase in SBP. In normal subjects, an augmentation of GCW was not observed along with changes in GWW and GWE, which indicated that the fluctuations of afterload in the physiological range led to an absolute increase in GCW but no changes in GWW and GWE. However, in people with hypertension, substantial augmentations of blood pressure not only increased the effective work but also caused an increment in the GWW. The increase in GWW may be related to the uncoordinated contractions caused by increasing wall stress. Since the increasing GCW could only partially offset GWW, a significant decline was observed in the GWE within the normal range.^14^ This finding reminds us that a larger increment in the afterload might diminish the efficacy of myocardial mechanics.

Compared to the controls, the changes in MW indices were higher in hypertensive patients. The possible reason for this may be significantly higher fluctuation of blood pressure in hypertensive patients than those of the controls. Along this line, we found that the changes in SBP, DBP, and MBP showed significant but weak correlations with the changes in MW indices. Elevated blood pressure can be balanced by the myocardial self‐regulation mechanism (Frank‐Starling mechanism, Anrep effect, and so on), but it may also increase the stiffness and the oxygen consumption of the myocardium. In the long run, chronically raised blood pressure may eventually promote myocardial fibrosis and LV remodeling, further causing LV failure. In hypertensive patients, instantly assessing the left ventricular function parameters may not be a sound clinical evaluation, and repeated observations of GLS and MW indices might be necessary.

### 4.1 Limitations

This study had several limitations. Firstly, it was single‐center research with a relatively small sample size. Secondly, although the pre‐existing left ventricular pathological changes such as hypertrophy, abnormal contraction, and dilatation may disturb the effect of changes in afterload, we could not perform a more detailed subgroup analysis since it was difficult with the limited sample size. In the subsequent work, we plan to confirm our results in a multicenter study with large sample size.

## 5 CONCLUSIONS

In healthy people without structural heart disease or cardiovascular risk factors, MW is relatively insusceptible to oscillations within the physiological range of blood pressure. However, in a population of hypertensive patients, a significant increment in blood pressure may lead to an increase in GWI and GCW to preserve left ventricular systolic function. Additionally, the increased GWW cannot be balanced by GCW, which results in a significant reduction of GWE. Repeated assessments of GLS and MW indices are necessary for hypertensive patients who display high fluctuations in blood pressure.

## AUTHOR CONTRIBUTIONS

Xinhao Li, Mei Zhang, Mengmeng Li, and Yu Zhang: conception, design, and analysis and interpretation of data. Xinhao Li, Quande Liu, Wuyun Bao, and Xiaoyu Wan: drafting of the manuscript or revising it critically for important intellectual content. Xinhao Li and Mei Zhang: final approval of the manuscript submitted.
